# Expanding the Single-Visit Approach for Cervical Cancer Prevention: Successes and Lessons From Burkina Faso

**DOI:** 10.9745/GHSP-D-17-00326

**Published:** 2018-06-27

**Authors:** Yacouba Ouedraogo, Gahan Furlane, Timothee Fruhauf, Ousmane Badolo, Moumouni Bonkoungou, Tsigue Pleah, Jean Lankoande, Isabelle Bicaba, Eva S. Bazant

**Affiliations:** a Jhpiego, Ouagadougou, Burkina Faso.; b Jhpiego, Baltimore, MD, USA.; c Johns Hopkins University School of Medicine, Baltimore, MD, USA.; d Société de Gynécologues et Obstétriciens du Burkina, Ouagadougou, Burkina Faso.; e Ministère de la Santé du Burkina Faso, Ouagadougou, Burkina Faso.

## Abstract

**Background:**

Cervical cancer accounts for 23% of cancer incidence and 22% of cancer mortality among women in Burkina Faso. These proportions are more than 2 and 5 times higher than those of developed countries, respectively. Before 2010, cervical cancer prevention (CECAP) services in Burkina Faso were limited to temporary screening campaigns.

**Program Description:**

Between September 2010 and August 2014, program implementers collaborated with the Ministry of Health and professional associations to implement a CECAP program focused on coupling visual inspection with acetic acid (VIA) for screening with same-day cryotherapy treatment for eligible women in 14 facilities. Women with larger lesions or lesions suspect for cancer were referred for loop electrosurgical excision procedure (LEEP). The program trained providers, raised awareness through demand generation activities, and strengthened monitoring capacity.

**Methods:**

Data on program activities, service provision, and programmatic lessons were analyzed. Three data collection tools, an individual client form, a client registry, and a monthly summary sheet, were used to track 3 key CECAP service indicators: number of women screened using VIA, proportion of women who screened VIA positive, and proportion of women screening VIA positive who received same-day cryotherapy.

**Results:**

Over 4 years, the program screened 13,999 women for cervical cancer using VIA; 8.9% screened positive; and 65.9% received cryotherapy in a single visit. The proportion receiving cryotherapy on the same day started at a high of 82% to 93% when services were provided free of charge, but dropped to 51% when a user fee of $10 was applied to cover the cost of supplies. After reducing the fee to $4 in November 2012, the proportion increased again to 78%. Implementation challenges included difficulties tracking referred patients, stock-outs of key supplies, difficulties with machine maintenance, and prohibitive user fees. Providers were trained to independently monitor services, identify gaps, and take corrective actions.

**Conclusions:**

Following dissemination of the results that demonstrated the acceptability and feasibility of the CECAP program, the Burkina Faso Ministry of Health included CECAP services in its minimum service delivery package in 2016. Essential components for such programs include provider training on VIA, cryotherapy, and LEEP; provider and patient demand generation; local equipment maintenance; consistent supply stocks; referral system for LEEP; non-prohibitive fees; and a monitoring data collection system.

## Abstract (precis)

The single-visit approach was implemented with strong attention to systems in 14 health facilities. In the 2 largest facilities, nearly 14,000 women screened for cervical cancer over 4 years. Of approximately 9% who screened positive, about 66% received same-day cryotherapy. Attention is needed to ensure local technicians can repair cryotherapy equipment, supplies are consistently in stock, and user fees are not prohibitive to accessing care.

Résumé en français à la fin de l'article.

## BACKGROUND

Globally, cervical cancer has the third highest incidence rate of all cancers in women, with more than 500,000 new cases per year, and causes an estimated 265,672 deaths per year.^1^ In Burkina Faso, the cervical cancer incidence and related mortality exceeds that of any other cancer in women,^1^ accounting for 23% of cancer incidence and 22% of cancer mortality. While these proportions are comparable with those of sub-Saharan Africa generally (25% incidence and 23% mortality) and Western Africa specifically (24% and 22%, respectively), they represent more than 2 times the incidence rate and 5 times the mortality rate of developed countries.^1^

In many developing countries, few skilled professionals are trained for cytology-based cervical cancer screening and surgical treatment of lesions, and the resources to sustain these costly services are also absent.^2^ Highly sensitive diagnostic and treatment tools including human papilloma virus testing and thermal coagulation require advanced health infrastructure and remain costly and vulnerable to loss of patients to follow-up.^3^^–^^5^ Visual inspection with diluted acetic acid (VIA) coupled with cryotherapy treatment for precancerous lesions is recommended by the World Health Organization (WHO)^6^ as an alternative, low-cost screening and treatment method. This single-visit approach (SVA) requires minimal infrastructure and can be practiced by non-physician health care providers following targeted training.^2^^,^^7^^,^^8^ In Burkina Faso, the Ministry of Health (MOH), professional associations, and various organizations have organized cervical cancer screening campaigns using VIA since the mid-2000s. However, treatment for precancerous lesions, including cryotherapy, had remained unavailable, leaving Burkina Faso's 1.7 million women ages 30–59 years without access to secondary prevention of cervical cancer.^9^^,^^10^

The single-visit approach to cervical cancer prevention requires minimal infrastructure and can be practiced by non-physician health care providers.

SVA has been implemented in several low-resource settings and its documented effectiveness has the potential to reduce cervical cancer mortality.^11^^,^^12^ Scale-up efforts are now underway in a number of countries, bridging screening and treatment services for cervical cancer prevention (CECAP).^12^^–^^14^ SVA is feasible, well-accepted, and safe despite initial concerns.^2^^,^^15^^,^^16^ However, debate remains regarding the best implementation approach for training providers,^2^^,^^14^^,^^17^ retaining providers,^14^^,^^18^^–^^20^ minimizing attrition through task shifting,^12^^,^^21^ and ensuring sustainability and integration of CECAP services into the health system.^2^^,^^7^^,^^12^^,^^20^^–^^22^ The wide spectrum of experiences in introducing SVA in several locations underlines the importance of adapting the implementation approach to the local context. In the interest of South-South collaboration, all experiences implementing SVA need to be widely shared so that programs can benefit from practical learning and CECAP services can be offered at scale.^2^^,^^7^^,^^23^

We describe the implementation of an integrated CECAP program in 14 health facilities, the challenges encountered, how they were overcome, and the outcomes of the program in the 2 teaching hospitals. Lessons for implementation and advocacy are drawn with the aim of benefiting other countries similarly expanding CECAP services.

## PROGRAM DESCRIPTION

Between September 2010 and August 2014, the project aimed to expand CECAP services in 14 health facilities at different levels in the health system throughout Burkina Faso by introducing VIA screening coupled with cryotherapy for precancerous lesions in a single visit. A second aim was to develop program monitoring skills among providers to foster independent and local improvement in service provision. The project's activities, described in the Table, encompassed training health care providers, demand generation, and capacity building in monitoring and evaluation.

**TABLE.** Objectives and Components of the CECAP Program in Burkina Faso

Objectives	Intervention	Activities	Challenges	Solutions
To strengthen institutional and provider capacity to provide CECAP services	Training	Provision of initial equipment for VIA, cryotherapy, and LEEP including parts and supplies Training of providers in counseling, VIA, cryotherapy, and LEEP Supportive supervision visits to mentor and support providers	Cryotherapy machine maintenance was performed abroad, reducing availability of services Shortage of supplies for VIA and cryotherapy (acetic acid, carbon dioxide, swabs, gauze)	Training local technicians to perform maintenance of cryotherapy machines internally Charging user fees to finance some of the costs of the procedures
To increase awareness about cervical cancer and CECAP services among providers and patients	Demand generation	Group education in facilities about cervical cancer and CECAP services Television programs about cervical cancer and CECAP services	Prohibitive user fees deterred demand for cryotherapy services at the same visit as screening	The implementing organization and SOGOB used a costing analysis to advocate for a reduced user fee in line with patients' financial capacity
To build local capacity to monitor program progress, identify shortcomings, and take corrective actions	Monitoring	Development of data collection tools: individual client form, client registry, and monthly summary sheet Training of data managers on data extraction and crosscheck methodology to improve data quality Training of providers on utilization of data to track program progress Routine review and dissemination of program results	Women referred from other facilities were screened and counted twice in program statistics	Tracking error was identified by providers and rectified in subsequent years to improve data quality

Abbreviations: CECAP, cervical cancer prevention; LEEP, loop electrosurgical excision procedures; SOGOB, Burkinabe Society of Gynecology and Obstetrics; VIA, visual inspection with acetic acid.

The single-visit approach was implemented in 14 health facilities in Burkina Faso between 2010 and 2014.

The CECAP program was started in 5 facilities as a collaborative project between the MOH and the implementing organization, Jhpiego, an American nonprofit organization focused on improving health systems and operating in more than 30 low-income countries. Through a partnership between Jhpiego and the Burkinabe Society of Gynecology and Obstetrics (SOGOB), additional resources were later leveraged from the Society of Obstetricians and Gynecologists of Canada and SEMAFO, a Canadian-based mining company, to expand CECAP services to all 9 regional hospitals. In total, 14 facilities were included in the program: 2 teaching hospitals (or Centre Hospitalier Universitaire, CHU), all 9 regional hospitals, and 3 district hospitals.

The CECAP program provided individual counseling to women ages 25 to 59. While cervical cancer screening programs typically start at age 30, local gynecologists favored the inclusion of women as young as 25 years old in this intervention because median age at sexual initiation is 17.7 in Burkina Faso.^24^ The program used a standard clinical protocol for VIA and cryotherapy based on the WHO guidelines for screening and treatment of precancerous cervical lesions and adapted to Burkina Faso's context through expert consultation.^25^ Women who screened negative by VIA received counseling and instructions to schedule a follow-up appointment in 3 years, unless they were living with HIV in which case they scheduled a follow-up appointment for repeat screening in 1 year. Women who screened positive by VIA and were eligible for cryotherapy received it at the time of the screening upon consenting to the procedure.

Eligibility for cryotherapy was based on WHO guidelines^6^:

1. The lesion is not suspicious of cancer.
2. The lesion does not extend into the endocervical canal.
3. The lesion occupies less than 75% of the cervix.
4. The cryotherapy machine tip (cryotip) covers the lesion (or less than 2 millimeters of the lesion extends beyond the edge of the cryotip).
5. The client is not pregnant.
6. The client is more than 12 weeks postpartum.

Women found to have larger lesions or lesions suspect for cancer were referred to the 2 university hospitals for loop electrosurgical excision procedure (LEEP) as part of the program's comprehensive secondary prevention services.

Women with larger lesions or lesions suspect for cancer were referred to the 2 university hospitals for LEEP.

### Training

We first trained 10 providers on VIA and cryotherapy in Malawi and Côte d'Ivoire during a 6-day training that gathered national training experts. Sessions were adapted to focus on the trainees' weaknesses identified through an initial assessment of baseline knowledge and skills. Training covered the general gynecologic exam, recognition of cervical landmarks and lesions, and interpretation of VIA results using photographic images. It also included practical training on conducting VIA and cryotherapy using anatomical models. These trained trainers then received technical assistance from Jhpiego to introduce SVA in their respective hospitals and train additional providers using the same 6-day training curriculum. The new trainers trained an additional 21 gynecologists, 2 general practitioners, and 27 nurse-midwives. Newly trained providers worked in pairs with a seasoned gynecologist or nurse-midwife who supervised their practice of VIA and cryotherapy for a minimum of 1 month. Eleven gynecologists were also trained to perform LEEP through a dedicated training organized by experts from Jhpiego.

### Demand Generation

Demand for cervical cancer prevention services was generated through organized educational activities. Every morning, trained midwives would facilitate informal educational discussions in the waiting areas for family planning and antenatal care services. Topics covered the female genital tract, the presentation of cervical cancer, cervical cancer screening, and available services. Service providers at the same facilities that were *not* trained in CECAP were also targeted with messages about the benefits of cancer screening and the introduction of SVA. In addition, trained providers received supportive supervision visits reinforcing SVA and CECAP service delivery standards, and a dedicated team of gynecologists and data managers ensured the quality of services through quarterly monitoring visits that followed Jhpiego's CECAP Monitoring and Evaluation Strengthening Guidelines. As part of the quality verification system, the gynecologists and data managers monitored inter-provider variation and reviewed pathology for some patients who underwent LEEP. Quality monitoring was particularly useful to identify providers over-diagnosing precancerous lesions and to define further training needs.

### Capacity Building in Monitoring and Evaluation

Besides service provision and demand generation, the program also built local capacity for CECAP service monitoring. Providers at the 2 highest-volume sites, the CHUs, were engaged in collecting data and improving data quality to monitor service delivery outcomes. Midwives designated as data managers were trained to extract data, crosscheck values, and make corrections using tools developed by the program. Jhpiego and SOGOB organized a quarterly review of service statistics and data quality checks: client forms, registries, and monthly summary sheets were crosschecked and discrepancies were discussed and corrected. Advice to improve data quality was shared with data managers. Providers were also trained to graph data on a laminated poster to display key indicators related to CECAP services. They used these charts to track the progress of program activities and disseminate results to program staff.

Program results were shared at national meetings that brought together program managers, SOGOB, other partners working in the realm of cervical cancer, and key decision makers including the MOH and the First Lady of Burkina Faso. Following these dissemination events, the government integrated CECAP into its national guidelines on cancer, *Plan stratégique de la lute contre le cancer 2013–2017*,^26^ thereby strengthening some of the program's achievements and sustainability.

## METHODS

The program collected 3 types of data: (1) program reports describing activities undertaken to introduce SVA and monitor results, (2) service records describing the number of clients reached by the program, and (3) implementation lessons learned via discussions with key stakeholders.

First, program reports were used to collect results of CECAP activities in the 14 health facilities between September 2010 and August 2014. Program reports, written each quarter, described progress against program objectives.

Second, the program developed 3 tools for monitoring service data: an individual client form, a client registry in which each client was listed, and a monthly summary sheet. Individual client forms completed by providers at each visit included client identification information, HIV status, VIA results, relevant treatment information, and follow-up plans. Program data managers extracted data from individual forms to populate client registries. Facility staff aggregated the registry data on the monthly summary sheet. These tools collecting individual- and facility-level data were used to track progress of 3 indicators:

- The number of women screened using VIA.
- The proportion of women who screened VIA positive.
- The proportion of women screening VIA positive who received same-day cryotherapy in a single visit approach (“proportion SVA”).

These indicators were disaggregated by HIV status according to Jhpiego's standard practice. HIV status was self-reported and extracted from the patient's medical booklet or assessed when the patient requested a voluntary HIV test. While this disaggregation was of interest for this program because of the increased risk for cervical cancer in women living with HIV, these numbers are not shown because Burkina Faso's HIV prevalence among women 15–49 years is 1.2%.^24^ Program staff and clinical providers reviewed data compiled every quarter. The authors collected and reviewed monitoring data from the 2 larger health facilities. SOGOB collected and reviewed monitoring data from the other 12 facilities and those data are not shown.

Third, formative lessons about the implementation of CECAP services were drawn from program experiences through discussions among program staff and with key stakeholders at the dissemination and advocacy events in Burkina Faso.

This program does not constitute a research study. Data collection and monitoring activities were carried out for quality improvement purposes and therefore were not subject to approval by an institutional review board. Privacy and confidentiality of personal information was maintained throughout data collection and analysis. All data kept by the program were de-identified.

## RESULTS

### Trained Providers

As mentioned previously, the program trained providers in VIA and cryotherapy through a partnership with programs in Malawi and Côte d'Ivoire. These trainers then trained 21 gynecologists, 2 general practitioners, and 21 nurse-midwives in Burkina Faso. In addition, 11 providers in the 2 CHUs were trained to provide LEEP for cases ineligible for cryotherapy. All providers continued to provide CECAP services over the course of the 4 years; no attrition was recorded. All SVA-trained providers were included in program monitoring workshops conducted throughout the 4-year implementation period; supportive supervision visits decreased in frequency over that time. Demand generation activities were conducted in all 14 facilities.

### Screened Clients

**FIGURE 1:**
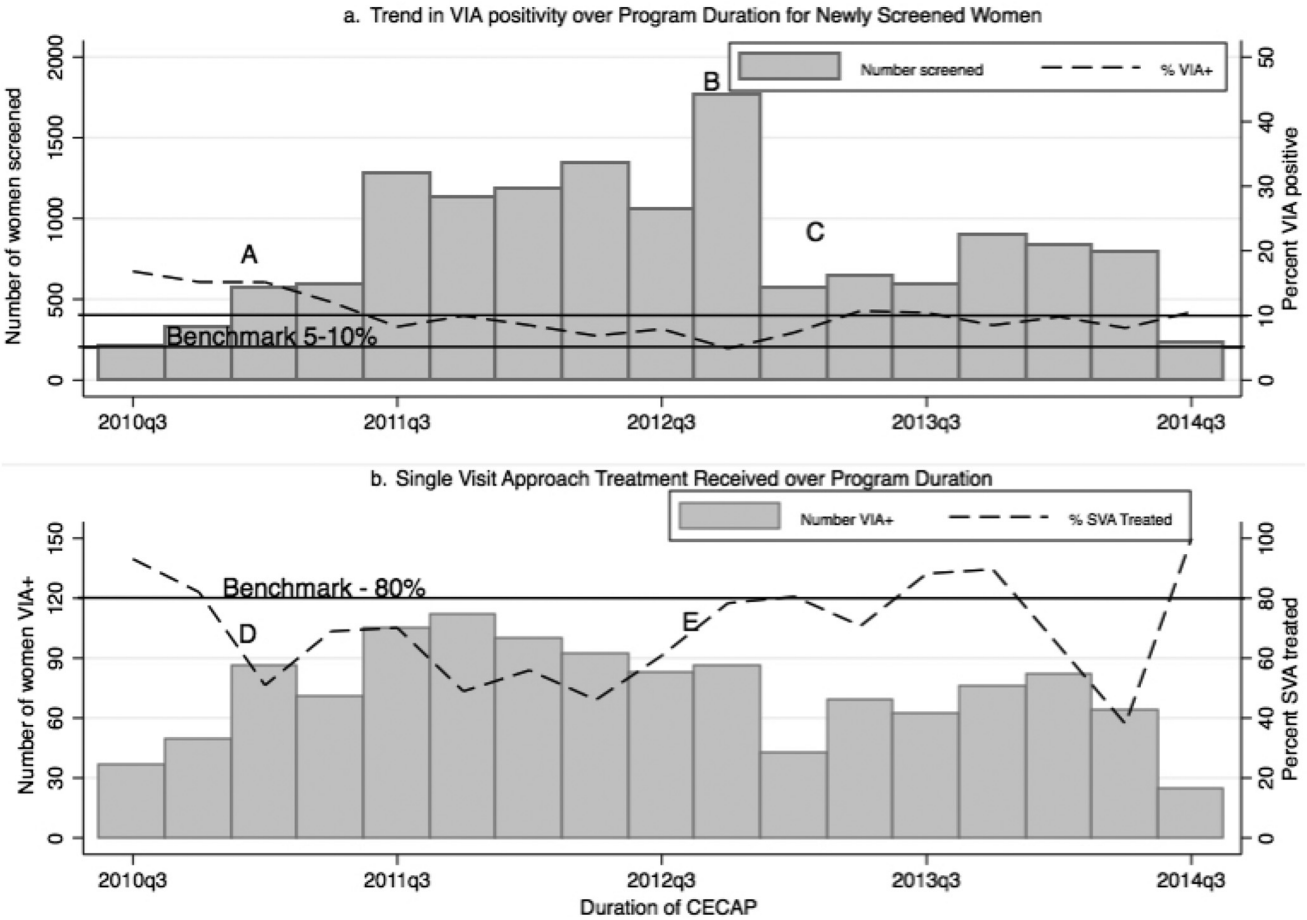
Trends in Cervical Cancer Screening, VIA Positivity, and Treatment, 2 Teaching Hospitals in Burkina Faso, September 2010–August 2014 Abbreviations: CECAP, cervical cancer prevention; SVA, single-visit approach; VIA, visual inspection with acetic acid. Notes: A. February 2011: Revision to the data collection tools to differentiate between clients referred for cryotherapy from other centers and clients making initial visits. B. October 2013: Television broadcast on cervical cancer by University Hospital Sorou Sanon. C. January–June 2013: Period without financial resources for supervision and procurement of consumables. D. January 2011: Facilities begin requiring payment for VIA and cryotherapy. E. November 2012: Cost reduction for cryotherapy.

Over the course of 4 years, 13,999 women were screened for cervical cancer using VIA. On average, 8.9% of the women screened positive by VIA. Several trends can be noted. First, the number of screened women increased dramatically with demand generation efforts in the first full year (2011) and second year (2012), from 2,713 to 4,662 (Figure 1a). The end of the initial funding phase led to a decrease in the number of women screened in the second quarter of the third year because there were fewer supportive supervision visits and several facilities experienced stock-outs of key supplies, including acetic acid, carbon dioxide, swabs, and gauze. Supportive supervision visits were less frequent but continued with assistance from SOGOB, the professional association.

Over 4 years, nearly 14,000 women were screened for cervical cancer and 9% screened positive.

Each quarter, 5% to 17% of women screened VIA positive (dotted line in Figure 1a). This proportion was in line with the international standard benchmark of 5% to 10%.^27^ The percentage was higher at the beginning of the program because some health centers did not offer cryotherapy and referred VIA-positive clients to CHUs where women were retested. These repeated screening results were recorded as though they were first diagnoses. This tracking error was rectified in the second year: these clients were logged as referrals from other health centers rather than new clients.

### Treated Clients

Of the 985 women who screened positive at first screening, 65.9% (649) received same-day cryotherapy treatment (Figure 1b). Additionally, 200 women received LEEP on a later date and 176 of these occurred in the 2 CHUs. Moreover, 151 women were referred for surgical management of suspected cancer. The proportion of VIA positive women who were treated with cryotherapy varied between 38% and 100% by quarter. This proportion was high (82% to 93%) at the start of the program when sites offered free cryotherapy because carbon dioxide gas was provided free of charge for the procedure. Later, when facilities were expected to support these costs, the SVA rate declined to 51% when a user fee for VIA and cryotherapy was charged to cover the cost of supplies. The prohibitively expensive fees (approximately $10 in a country where 44.5% of the population was living on less than $1.25 per day in 2009^28^) led many women to postpone cryotherapy.

Of those who screened positive, 66% received same-day treatment with cryotherapy.

The increase in the proportion of VIA positive women treated with cryotherapy in late 2012 may have been linked to two changes. First, program implementers and SOGOB advocated for lower prices to the MOH and facilities management teams. In November 2012, after the cryotherapy fee was reduced to $4 in CHUs, the proportion of women receiving cryotherapy increased again to 78%. Second, the capacity to repair cryotherapy machines improved locally. Treatment services had typically been intermittently halted when broken cryotherapy machines were sent out of the country for repairs. In 2012, the program hired the cryotherapy machine manufacturer to train 12 onsite technicians from the health facilities to repair machines. Maintenance and repairs were then conducted onsite reducing hiatuses in treatment provision.

### Midcourse Corrections Based on Use of Program Monitoring Data

Providers collected data on services through the 3 tools—client forms, the client registry and monthly summary sheets—allowing the onsite managers and providers themselves to visualize progress, analyze trends, and identify potential bottlenecks in service provision. Displaying key indicators on posters meant providers had the tools to evaluate themselves and take corrective actions in real time. For instance, providers noticed that the proportion of women who screened positive by VIA was higher than expected and identified the source to be the incorrect recording of referred patients. Women with positive VIA tests at outside facilities who were referred for cryotherapy had a repeat VIA test prior to cryotherapy leading referred patients to be counted twice as having positive VIA tests. By tracking indicators, they also realized that the fees charged to patients were too high and gathered evidence to effectively convince facility managers to reduce these costs.

## DISCUSSION

The program introduced comprehensive CECAP services through same-day VIA screening and cryotherapy treatment for eligible women in 14 health facilities in Burkina Faso through the combined efforts of several institutions. The program had several components: (1) training providers to engage in same-day screening using VIA and cryotherapy for treatment, and referral for and performance of LEEP for women with larger lesions or lesions suspect for cancer, (2) generating demand for cervical cancer prevention services among patients and providers, and (3) building capacity among providers to collect and use monitoring data to track progress and take corrective actions locally. Over 4 years, this approach allowed the program to screen 13,999 women for cervical cancer, detect 8.9% of VIA-positivity among women screened, and treat 65.9% of the women testing VIA-positive in a single visit. The 80% treatment target set by the program was reached at the start of the program when services were provided free of charge, but once the program started charging treatment fees to cover the cost of supplies, the proportion treated in a single visit dropped to 51% because the user fees were prohibitive. Once the fees were lowered from $10 to $4, the proportion treated in a single visit increased again to 78%.

Once the program started charging treatment fees, the proportion of patients treated in a single visit dropped, from over 80% to 51%. When the fees were lowered, this proportion increased to 78%.

Overall, the program increased awareness of cervical cancer prevention services among patients, leading to service uptake from previous levels of zero (as services were not offered before this program). Program uptake suggests the acceptability and feasibility of such a program in Burkina Faso in the context of donor funding. Following the dissemination of the program's results, the MOH integrated CECAP services into its strategic planning document, laying the way forward for possible future expansion of comprehensive CECAP services in Burkina Faso. Since then, all health districts have included CECAP services in their annual action plans, especially with regard to provider training. The MOH is developing strategies to overcome gaps in availability of cryotherapy equipment.

The MOH integrated cervical cancer prevention services into its strategic planning document, laying the way forward for possible future expansion in the country.

Challenges related to implementation occurred. First, referred women who received a repeat screening were initially counted twice when tracking the provision of services; this was later corrected by including referral status in the registries. Second, some facilities could not provide CECAP services continuously because there were shortages of supplies necessary for VIA and cryotherapy. In addition, sometimes cryotherapy machines were unavailable when they were being repaired in other countries. Training local technicians to perform maintenance internally in the facilities and charging fees to finance some of the costs of the procedure addressed some of these issues. Third, fees were initially set at a prohibitive level for the patient population and deterred use of the services. SOGOB negotiated a lowering of these fees to an appropriate level.

This program experience confirms many aspects of SVA implementation detailed in the literature. For example, data monitoring and quality checks have been beneficial to CECAP service provision in published experiences.^12^^,^^20^^,^^21^^,^^29^^–^^31^ However, only this Burkina Faso program has integrated that aspect in a framework of capacity building and placed local providers at the center of using that data for problem and solution identification. Other programs also noted the importance of continued supervision to guarantee quality outcomes^8^^,^^12^^,^^32^ and access to consistent supplies and operational equipment.^5^^,^^12^^,^^30^^,^^33^ The literature also reports on the difficulty of maintaining the screen-and-treat continuum by minimizing loss to follow-up and ensuring that women return for cryotherapy if the SVA is not possible for a patient.^14^^,^^22^^,^^33^ This program identified user cost as a key barrier to follow-up and successfully addressed it. Finally, support from the MOH was also found to be instrumental in incorporating CECAP services into the health agenda and scaling up services in Botswana, Guyana, Mozambique, Tanzania, and Zambia.^20^^,^^21^^,^^32^^,^^34^^–^^36^

### Essential Components and Lessons Learned

In light of these challenges faced by the Burkina Faso program and those recounted in the literature, essential components for the establishment of a national CECAP program include:

1. Adequate training of physicians and midlevel providers, including nurses and nurse-midwives, accompanied by a quality assurance protocol.
2. Effective demand generation campaigns targeting patients and providers.
3. Local cryotherapy equipment maintenance.
4. Consistent stocks of VIA and cryotherapy supplies.
5. A referral system for large lesions that need LEEP and suspect cancer cases.
6. Non-prohibitive fees that allow access to services.
7. A robust routine monitoring data collection system used to identify and address service delivery gaps.

Some components are likely setting-specific and should be adapted to consider local contexts.

Three key lessons can be drawn from the implementation of this CECAP program. First, fostering champions of cervical cancer prevention was essential to influence stakeholders and decision makers to increase their commitment to CECAP services. This program nurtured these reproductive health champions and trained them to adopt and promote SVA for cervical cancer. They trained additional providers, which expanded the reach of the program. Their leadership led to increased interest in CECAP services not only among other medical professionals, including obstetricians-gynecologists, nurses and midwives, and SOGOB generally, but also among political figures and experts at the MOH.

Second, the collection, visualization, and display of routine monitoring data in real time by the providers themselves empowered them to track progress, identify gaps, and take corrective actions to remedy any shortcomings, thereby reaching more successful outcomes. The program's focus on strengthening the monitoring capacity of local providers enabled them to become participants in the program's success and essential players in not only identifying issues themselves but also conceptualizing innovative solutions. For instance, through these monitoring data, providers were actively engaged in brainstorming solutions about tracking women referred to other facilities to report accurate indicators, reducing the cost of cryotherapy for patients, and improving internal machine maintenance capacity.

Third, stakeholder coordination under the umbrella of national guidelines is essential to ensure the growth and sustainability of a cervical cancer prevention program focused on increasing the provision of services. Prior to 2013, there was no national program dedicated to CECAP and different stakeholders lacked coordination: local associations conducted separate outreach campaigns for cervical cancer screening without linking screening to timely and appropriate treatment in a facility. Providers did not have the technical support they needed to introduce comprehensive CECAP services. Supportive supervision from district management teams to health facilities did not include CECAP, and the health management information system did not collect data on CECAP. Through training of trainers, technical assistance, and capacity building on monitoring, this program began to coordinate the different players with a role in the provision of CECAP services among the 14 facilities where it was implemented.

Gathering key decision makers for the dissemination of the program's results and engaging them in the successes, shortcomings, and revisions of the experience illustrated not only the importance of cervical cancer prevention services in Burkina Faso but also the acceptability, feasibility, and impact of providing these services. This process is believed to be essential to the sustainability of providing CECAP services. Since the program's end, CECAP services have continued to be offered routinely in the 14 sites and supplies and materials for these activities have been included in the facilities' budgets.

These dissemination efforts were also crucial for sustainability at the national level. Advocacy activities conducted with SOGOB lent support to CECAP services and promoted the integration of VIA-based screening and cryotherapy in the national plan for cancer control. In March 2016, the MOH issued a decree making cervical and breast cancer screening and basic treatment free of charge. In April 2016, CECAP became part of the minimum package of services offered to women in facilities in Burkina Faso. Through these commitments, Burkina Faso has progressively become a model for the successful integration of cervical cancer prevention efforts in resource-limited settings and illustrated a path toward implementing a part of the global cancer-free agenda.

## Notes

**En français**

**Etendre l'approche de la visite unique pour la prévention du cancer du col de l'utérus : succès et leçons apprises du Burkina Faso**

L'approche de la visite unique a été mise en œuvre avec une attention particulière au renforcement du système dans 14 formations sanitaires. Dans les deux plus grandes structures sanitaires, près de 14 000 femmes ont bénéficié d’un dépistage des lésions précancéreuses du col de l'utérus en quatre ans. Sur environ 9% de cas positifs, environ 66% ont reçu la cryothérapie le jour même. Une attention particulière est nécessaire pour s'assurer que les techniciens locaux peuvent réparer les équipements de cryothérapie, que les produits sont disponibles et que les tarifs pratiqués pour l’offre de service ne constituent pas une barrière a l'accès aux soins.

**RÉSUMÉ**

**Contexte :** Le cancer du col de l'utérus représente 23% de l'incidence du cancer et 22% de la mortalité par cancer chez les femmes au Burkina Faso. Ces proportions sont respectivement plus de 2 et 5 fois plus élevées que celles des pays développés. Avant 2010, les services de prévention du cancer du col de l'utérus (CECAP) au Burkina Faso se limitaient à des campagnes de dépistage sporadiques.

**Description du programme : **Entre septembre 2010 et août 2014, une équipe de Jhpiego a collaboré avec le ministère de la Santé et les associations professionnelles pour mettre en œuvre un programme CECAP axé sur le couplage de l'inspection visuelle du col a l'acide acétique pour le dépistage et le traitement par cryothérapie des lésions précancéreuses chez des femmes éligibles dans 14 formations sanitaires. Les femmes avec des lésions plus grandes ou des lésions suspectes de cancer ont été référées pour la résection a l’anse diathermique (RAD). Le programme a formé des prestataires, conduit des activités de sensibilisation pour la création de la demande et renforcé la capacité en suivi.

**Méthodes : **Les données sur les activités de programme, les prestations de services et les leçons programmatiques ont été analysées. Trois outils de collecte de données à savoir un formulaire individuel client, un registre client et une fiche de synthèse mensuelle ont été utilisés pour suivre trois principaux indicateurs de service CECAP : nombre de femmes dépistées à l'aide de l’IVA, proportion de femmes dépistées positives a l’IVA et proportion de femmes dépistées positives à l’IVA qui a reçu la cryothérapie le jour même du dépistage.

**Résultats : **En 4 ans, le programme a dépisté 13 999 femmes pour les lésions précancéreuses du col de l'utérus en utilisant l’IVA; 8,9% ont été dépistés positives; et 65,9% d’entre elles ont reçu une cryothérapie au cours de la même visite. La proportion de patients recevant la cryothérapie le même jour est passée de 82% à 93% lorsque les services étaient fournis gratuitement, mais est tombée à 51% lorsque des tarifs d’environ 10 $ ont été appliqués pour couvrir le coût des prestations. Après avoir réduit les frais à environ 4 $ en novembre 2012, la proportion a encore augmenté pour atteindre 78%. Les difficultés de mise en œuvre comprenaient celles du suivi des patients référés, les ruptures de stock de produits essentiels, les difficultés de maintenance des machines de cryothérapie et les tarifs prohibitifs appliqués pour les prestations de service. Les prestataires ont été formés pour assurer de manière autonome le suivi des indicateurs d’offre de services, identifier les lacunes et prendre des mesures correctives.

**Conclusions : **Après la diffusion des résultats démontrant l'acceptabilité et la faisabilité du programme CECAP, le ministère de la Santé du Burkina Faso a inclus les services CECAP dans son paquet de prestation de services minimum en 2016. Les composantes essentielles de ces types de programmes comprennent la formation des prestataires sur l’IVA, la cryothérapie et la résection a l’anse diathermique; la création de la demande des services au niveau des prestataires et des clients; la maintenance des équipements au niveau local; un approvisionnement adéquat en produits; un système de référence pour la RAD; des tarifs non prohibitifs et un système de collecte de données performant.

## Notes

**Peer Reviewed**

**Funding:** Izumi Foundation
